# Imaging of Nitric Oxide in Nitrergic Neuromuscular Neurotransmission in the Gut

**DOI:** 10.1371/journal.pone.0004990

**Published:** 2009-04-02

**Authors:** Hemant S. Thatte, Xue D. He, Raj K. Goyal

**Affiliations:** 1 Department of Surgery (Cardiothoracic Division), VA Boston Health Care System and Harvard Medical School, Boston, Massachusetts, United States of America; 2 Center for Swallowing and Motility Disorders, Departments of Medicine, VA Boston Health Care System and Harvard Medical School, Boston, Massachusetts, United States of America; Hospital Vall d'Hebron, Spain

## Abstract

**Background:**

Numerous functional studies have shown that nitrergic neurotransmission plays a central role in peristalsis and sphincter relaxation throughout the gut and impaired nitrergic neurotransmission has been implicated in clinical disorders of all parts of the gut. However, the role of nitric oxide (NO) as a neurotransmitter continues to be controversial because: 1) the cellular site of production during neurotransmission is not well established; 2) NO may interacts with other inhibitory neurotransmitter candidates, making it difficult to understand its precise role.

**Methodology/Principal Findings:**

Imaging NO can help resolve many of the controversies regarding the role of NO in nitrergic neurotransmission. Imaging of NO and its cellular site of production is now possible. NO forms quantifiable fluorescent compound with diaminofluorescein (DAF) and allows imaging of NO with good specificity and sensitivity in living cells. In this report we describe visualization and regulation of NO and calcium (Ca^2+^) in the myenteric nerve varicosities during neurotransmission using multiphoton microscopy. Our results in mice gastric muscle strips provide visual proof that NO is produced *de novo* in the nitrergic nerve varicosities upon nonadrenergic noncholinergic (NANC) nerve stimulation. These studies show that NO is a neurotransmitter rather than a mediator. Changes in NO production in response to various pharmacological treatments correlated well with changes in slow inhibitory junction potential of smooth muscles.

**Conclusions/Significance:**

Dual imaging and electrophysiologic studies provide visual proof that during nitrergic neurotransmission NO is produced in the nerve terminals. Such studies may help define whether NO production or its signaling pathway is responsible for impaired nitrergic neurotransmission in pathological states.

## Introduction

Nitric oxide (NO) has been proposed as a neuromuscular neurotransmitter of nonadrenergic noncholinergic (NANC) inhibitory nerves in the parasympathetic [1] and the enteric nervous systems [2]. Clinical importance of this signaling pathway is evidenced by the fact that animal models of impaired nitrergic neurotransmission reveal phenotypes resembling major human gastrointestinal motility disorders [3]–[7]. However, because of the unusual characteristics of NO, its status as a neurotransmitter or its regulation remains unsettled.

While there is strong physiological evidence that NO is involved in inhibitory neurotransmission [8]–[9], its role as a true neurotransmitter has been questioned. It has been argued that NO may a mediator of another neurotransmitter such as VIP [10]–[11]. This later view is supported by biochemical studies in the isolated postjunctional smooth muscle cells showing that VIP generates NO in the smooth muscles [12]. However, in a critical review of the available evidence, Van Geldre and Lefebvre [11] concluded that VIP-generated NO in the isolated smooth muscles may be nonphysiological. Based on electrophysiological studies, it has been proposed that during nitrergic neurotransmission, NO is generated in the nerves and is a true inhibitory neurotransmitter [13]–[14].

NO has a unique chemistry. It reacts with 4,5-diaminofluorescein (DAF-2) to produce quantifiable fluorescent product DAF-2 triazole (DAF-2T). Reaction of NO with DAF-2 is highly specific and sensitive (NO detection limit of 5 nM) [15]–[19] and can be simultaneously imaged with Ca^2+^ [20]. DAF-2 is not toxic to living cells and does not impair cellular function [17]–[18]. Imaging performed with a multiphoton microscopy [20]–[22] can visualize the myenteric nerve varicosities and other structures, including smooth muscle cells, at deeper levels below the surface.

The purpose of the present studies was to examine the effect of electrical field stimulation of mice gastric muscle strips: 1) on NO and Ca^2+^ signals at the cellular level by fluorescent imaging; 2) on the effect of pharmacological treatments on these signals; 3) on nitrergic slow inhibitory junction potential (sIJP) in electrophysiological studies; and 4) to compare the effects of the pharmacological treatments on NO in the imaging studies and sIJP in the electrophysiological studies.

These results provide, for the first time, visual identification of nerve varicosities *in situ* in the gut and also provides proof that on NANC nerve stimulation, NO is produced in the myenteric nitrergic nerve varicosities and not in the smooth muscle cells, thereby demonstrating that NO is a neurotransmitter rather than a mediator produced in the smooth muscles. They also document that during neurotransmission NO is produced *de novo* and not stored as a NO donor in the varicosities and released with other classical neurotransmitters. Changes in NO production in response to various pharmacological treatments correlated well with changes in slow inhibitory junction potential of smooth muscles. Functional studies combined with imaging may help elucidate whether NO production or its upstream or downstream signaling is the underlying mechanisms of impaired nitrergic neurotransmission in the pathological states.

## Results and Discussion

### Visualization of myenteric nerve varicosities by their Ca^2+^ signals

**Figure 1:**
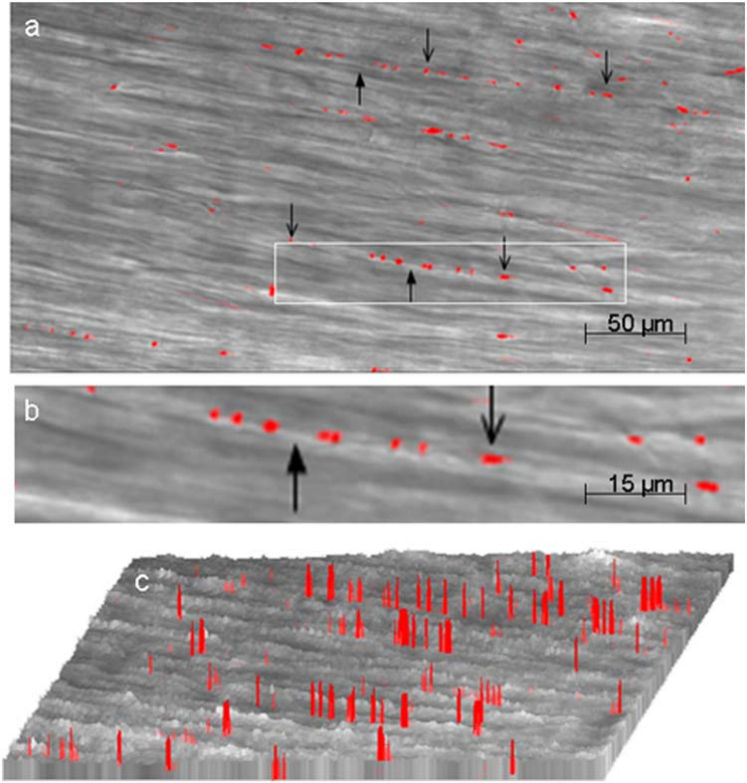
Visualization of myenteric nerve varicosities by their Ca^2+^ signals. These studies were done after EFS of the muscle strips preloaded with the indicator calcium orange. The fluorescent image was superimposed on the image in the transmission mode (a) is a low power view fluorescent (orange-red) varicosities. Note the varicosities along the axon running in the long axis of the circular muscles. (b) is magnified view of one of the axons with varicosities along its length . Downward pointing arrows show the varicosities and upward pointing arrows indicate the inter-varicosity axon. (c) shows intensity profiles of the fluorescent varicosities. Note that there was no Ca^+^ signal in smooth muscle cells.

We first sought to visualize varicosities of myenteric neurons in the mouse gastric smooth muscle. Imaging was focused on the varicosities because they are the sites of release of the neurotransmitters. Multiphoton imaging of circular muscle strips preloaded with the calcium indicator after EFS revealed discrete orange-red fluorescent spots. These images were superimposed on image of smooth muscles obtained in the transmission mode. Note that varicosities were linearly oriented along the longitudinal axis of the underlying muscle fibers (Figure 1). These fluorescent spots were not seen in tissues pretreated with tetrodotoxin, suggesting that they represented nerve varicosities. Figure 1a shows a low power view of the varicosities visible against the background of the non-fluorescent smooth muscle cells at a depth of 150 µm from the surface of the strip. Varicosities appeared as pearl-like structures that are linearly arranged along the axis of the underlying smooth muscle cells. These experiments were performed under NANC conditions that blocked neural excitation of the smooth muscles. Figure 1b shows a magnified view of an axon with the varicosities. The varicosities varied somewhat in their size and were on an average 2–4 µm×2–3 µm and were separated from each other, with inter-varicosity interval varying from 2 µm to greater than 200 µm. Figure 1c shows intensity (height) and width of localized fluorescent calcium signals. These columns represent fluorescent signals from the varicosities. This view also shows that the varicosities are linearly arranged on an axon and are separated by inter-varicosity intervals. Ca^2+^ signals identify all nitrergic and non-nitrergic varicosities. These studies show that multiphoton microscopy can vividly visualize varicosities on axons deep below the surface in intact tissue. Elevated Ca^2+^ signals were not seen in smooth muscles because EFS was applied under nonadrenergic noncholinergic conditions to block muscle excitation.

### Visualization of NO in the varicosities

**Figure 2:**
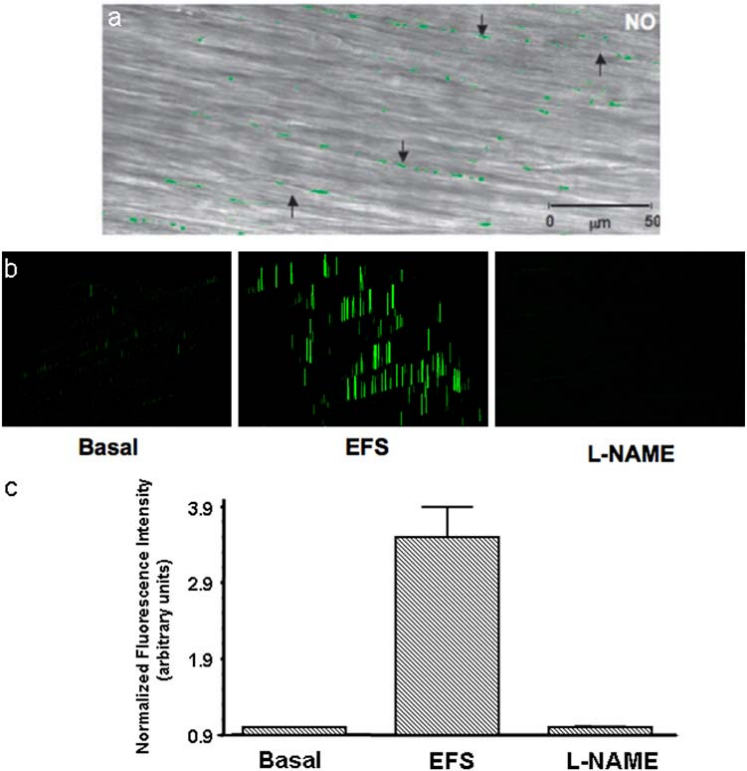
Visualization of NO signals in the varicosities. These studies were done after EFS of the muscle strips preloaded with NO indicator DAF-2. (a) is a low power view showing the superimposed NO fluorescent image and the image in the transmission mode. Note the green fluorescent varicosities seen along axons running in the long axis of smooth muscles. Also note that there were no NO signals in the smooth muscles. (b) Represents examples of intensity profiles of the NO signals in the unstimulated varicosities, the varicosities in the muscle strip stimulated with EFS and a muscle strip that was treated with L-NAME prior to the stimulus. (c) Represents relative quantification of the NO signals. Note very small NO signals in the unstimulated state and in stimulated state after L-NAME treatment.

To visualize nitrergic varicosities, we examined muscle strips preloaded with DAF-2 after applying EFS under NANC conditions. Green DAF-2T fluorescence represent NO signals (Figure 2). Panel (2a) shows fluorescent green NO signals in nitrergic varicosities superimposed on the underlying smooth muscle layer imaged in the regular transmission mode. Note the absence of NO signals in the smooth muscle cells. The neurally released NO may diffuse into the postjunctional smooth muscles or ICCs to exert its effects on these structures. However, no NO signals were seen in the smooth muscles or ICCs, suggesting that the level of NO in the target tissue was below the threshold of detection and may have been consumed by its action on the target enzymes.

We also examined NO signals in the strips preloaded with DAF-2DA but not electrically stimulated, tissues that received electrical stimulation and the tissue that were pretreated with L-NAME prior to EFS. Panel (2b) shows intensity (height and width) of the NO fluorescent signals from the varicosities. Note that very few NO signals were seen in the strips without EFS. The signals increased in the strips that received EFS and were again absent in the strips that receive EFS after L-NAME treatment. Panel (2c) shows relative quantification of the NO signals. The bar graphs revealed that NO signal was 1.5±0.25 in the basal state (unstimulated strips), 4.0±0.97 after EFS, and 1.0±0.03 in strips pretreated with NOS inhibitor, L-NAME (mean±SEM of normalized fluorescence intensity in arbitrary units, n = 6). Basal levels of NO may be generated by the tonic activity of the nitrergic neurons. These observations strongly suggest that the green signals are truly due to NO produced in the nerve varicosities.

### Colocalization of NO and Ca^2+^ in the varicosities

**Figure 3:**
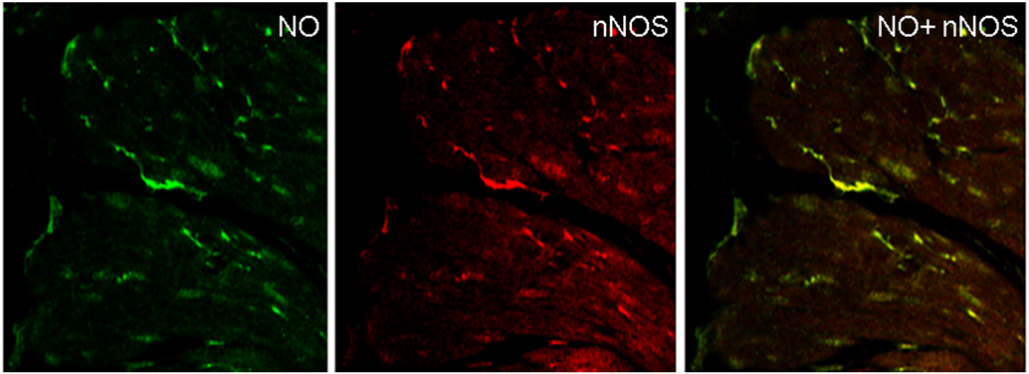
Colocalization of NO and Ca^2+^ in the varicosities. The muscle strip simultaneously preloaded with calcium orange and DAF-2A were electrically stimulated and the varicosities were examined for Ca^2+^ and NO signals. Top panel shows green NO positive varicosities. Middle panel shows orange-red calcium positive varicosities. Bottom panel shows colocalized NO and Ca^2+^ signals. Neither NO nor Ca^2+^signals were seen in the neighboring smooth muscle cells. (320× magnification).

In order to identify whether NO signals were produced in prejunctional nitrergic nerve terminals that also showed Ca**^2+^** signals, we loaded the muscle strips with both DAF-2 and calcium orange and applied EFS. These strips were imaged for NO and Ca^2+^ signals (Figure 3). Top panel shows green NO signals and middle panel shows orange-red Ca^2+^ in the varicosities. Bottom panel shows yellow color of the colocalized Ca^2+^ and NO signals. Some varicosities showed only orange-red fluorescence without yellow fluorescence; these may represent non-nitrergic varicosities. Preliminary studies of serial 1 second imaging of calcium and NO signals showed that Ca^2+^ signal appeared within 1 second of EFS and the NO signal followed it. Further dynamic studies using a calcium dye with fast kinetics are needed to fully document temporal relationship of the Ca^2+^ and NO signals.

### Colocalization of NO and nNOS in the varicosities

**Figure 4:**
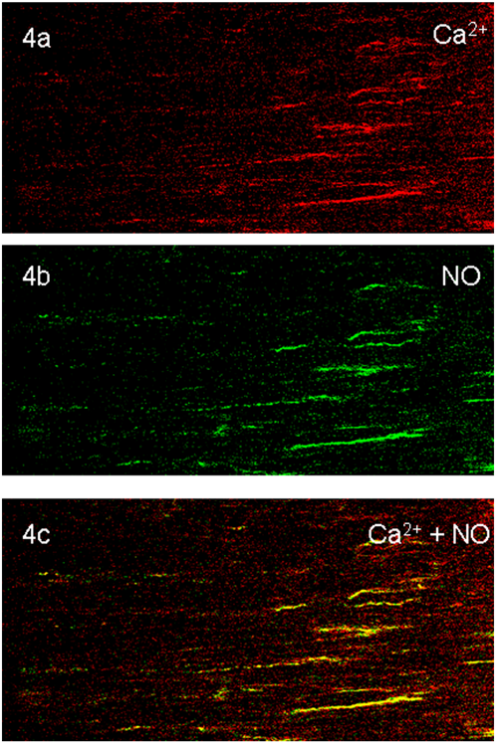
Colocalization NO signals with nNOS immunoreactive varicosities. In this experiment the muscle strip that was preloaded with DAF-2 under NANC conditions, applied EFS and then fixed was immunostained with anti-nNOS antibody. The NO signals are long lasting as the reaction of NO with DAF-2 to form DAF-2T is irreversible. Top panel shows imaging for NO and reveals spots of green fluorescence, representing NO in the varicosities. Note that smooth muscle cells showed no NO signals. The middle panel shows the same section imaged for nNOS immunofluorescence. The bottom panel shows the merged image revealing that the NO and nNOS signals are superimposed indicating that NO was generated in the nNOS immunoreactive nerves. Note that no NO signals were seen in neighboring smooth muscle cells. (320× magnification).

In order to identify whether NO signals were produced in prejunctional nitrergic nerve terminals, we applied EFS to the tissues that had been loaded with DAF-2. Since the reaction of DAF-2 with NO is irreversible, the fluorescent DAF-2T marker remained in the varicosities for a long time. These strips were then immunostained with anti-nNOS antibody. The muscles strips with DAF-2T marker and anti-nNOS staining were examined for colocalized fluorescence. NO signals were colocalized to the nerve terminals that showed immunoreactivity to nNOS, indicating that NO production occurred in the nitrergic nerve varicosities (Figure 4). These imaging studies provide visual proof that during nitrergic neurotransmission, nitric oxide is produced *de novo* in the nitrergic nerve varicosities. NO signals were not seen in the smooth muscle cells.

### Effect of various antagonists on Ca^2+^ and NO signals in the muscle strips

We also examined the effect of various known antagonists of nitrergic neurotransmission on Ca^2+^ and NO signals in the electrically stimulated strips preloaded with DAF-2 and calcium orange. Table 1 summarizes the relative quantification of NO and Ca^2+^ signals after various antagonist treatments. Note that EFS (control) increased NO and Ca^2+^ signals. The elevation of NO and Ca^2+^ signals were abolished by tetrodotoxin. Since tetrodotoxin blocks the fast sodium channel that mediates the action potential that is conducted along the axon and depolarizes the nerve varicosities to cause Ca^2+^ influx [11], these results suggest that the EFS response was due to stimulation of cell bodies or fiber tracts rather than direct stimulation of the varicosities. Effect of EFS was blocked by the selective inhibitor of N-type Ca^2+^ channels, ω-CTX GVIA, so that no significant increase in Ca^2+^ or NO signals were seen. However, L-type Ca^2+^ channel blocker, nifedipine, did not alter the increases in Ca^2+^ or NO signals. These observations indicate that Ca^2+^ entry into the varicosities that stimulates NO production occurred via N-type Ca^2+^ channels. Pretreatment of tissues with calmodulin (CaM) inhibitor W7 did not affect Ca^2+^ increase, but markedly suppressed NO production by EFS , suggesting that increase in internal Ca^2+^ stimulates nNOS via a Ca^2+^-CaM mediated process to produce NO. Similarly, pretreatment with the nNOS inhibitor L-NAME suppressed NO signals without affecting the Ca^2+^ signals, showing that suppression of nNOS caused suppression of NO generation in the presence of normal increase in Ca^2+^ upon electrical stimulation.

**Table 1:** Influence of various treatments on relative quantification of NO and Ca^2+^ signals.

Treatment	NO signal	Ca^2+^ signal
Control	4.0±0.97	10.5±1.30
TTX	1.0±0.01	1.0±0.01
ω-CTX GVIA	1.0±0.01	1.0±0.01
Nifedipine	4.7±1.50	9.7±2.00
W7	1.0±0.01	10.5±1.80
L-NAME	1.0±0.03	9.1±0.63

Values represent mean±SEM of normalized fluorescence intensity in arbitrary units after EFS from three independent experiments.

### Effect of various antagonists on the slow IJPs in mice gastric muscle strips

**Figure 5:**
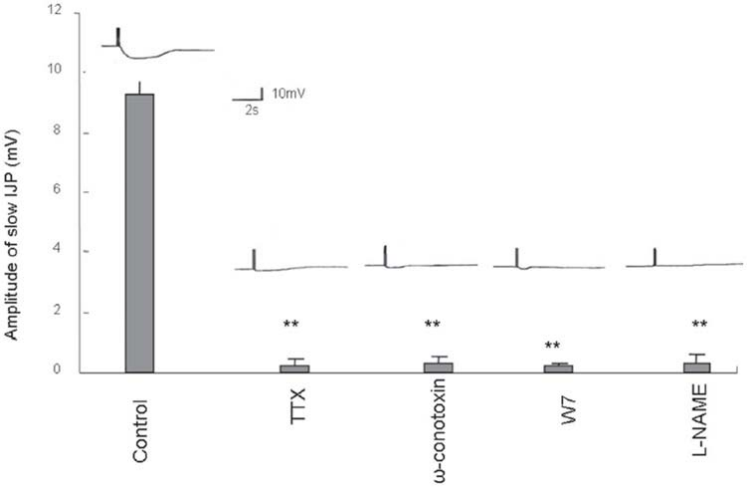
Effect of various antagonists on the slow IJPs in mice gastric muscle strips. The slow IJP was produced in response to EFS under NANC conditions. Note that the slow IJP was suppressed by pretreatment with TTX and ω-CTX GVIA but nifedipine had no effect (data not shown). Moreover, calmodulin antagonist, W7 and nNOS inhibitor, L-NAME also blocked the slow IJP. Representative sIJPs are shown at the top of the bar of the respective treatment groups.

In order to correlate pharmacology of imaging studies with functional neurophysiological studies of smooth muscle membrane potentials, we examined the effects of antagonists on the nitrergic slow inhibitory junction potentials (sIJP). EFS of muscle strips under NANC conditions produced two overlapping IJPs called the fast and the slow IJPs. Apamin treatment blocked fast IJP and revealed the nitrergic slow IJP [13], [14], [23]. The slow IJP was blocked by TTX, ω-CTX GVIA as well as W7 and L-NAME, but was not affected by apamin or nifedipine. Figure 5 shows representative slow IJP and summarizes the quantitative data. Bars represent mean values±SEM (6 cells, n = 3 mice). These results show that antagonists of physiologic nitrergic slow IJP also suppress NO signals in the varicosities and these events can be documented using the imaging studies.

### Conclusions

In conclusion, the unique chemical properties of NO and its indicator dyes and multiphoton microscopy allows imaging of NO during nitrergic neurotransmission that is not possible with many other neurotransmitters. These studies provide visual proof that NO is a true neurotransmitter and not a secondary mediator.

Imaging of nitrergic neurotransmission may help distinguish between disorders due to impaired NO production such as nNOS deficiency and those due to impaired NO action such as seen in deficiencies of NO sensitive guanylyl cyclase [4], cGMP kinase1 [5], Collagen XIXa1 [6] or c-kit [24]. Simultaneous NO and Ca^2+^ imaging studies combined with neurophysiology may also provide an important tool for understanding mechanisms of impaired nitrergic neurotransmission in motor disorders of the gut. Such studies may also help better define the underlying defect in nitrergic neurotransmission in conditions such as diabetic gastroparesis [7] and other human gastrointestinal diseases like achalasia [6] and abnormal gastrointestinal motility due to undefined cause. Simultaneous imaging of Ca^2+^ and NO can also help document whether the suppressed nitrergic neurotransmission is due to abnormalities in calcium kinetics, CaM abnormalities or defects in the enzyme, nNOSα. Such studies may also be helpful in elucidating abnormalities in urinary tract and cerebral blood vessels where nitrergic neurotransmission is a major regulatory mechanism.

## Materials and Methods

### Ethics Statement

The experimental protocol used was approved by the Animal Care Committee of the VA Boston Healthcare System.

### Animals and Tissue Preparation

CO_2_ narcosis was used to euthanize adult male mice (22–38 g). Stomach was removed and 4–6 mm wide strips of smooth muscle layer were prepared after shearing the mucosa. The strips were transferred to a tissue bath with a Sylgard (Dow-Corning, Midland, MI) floor and pinned to the floor with mucosal surface facing up. The chamber was continuously perfused with warm oxygenated (95% O_2_/5% CO_2_) Krebs solution at a rate of 3 ml/min. The bath temperature was maintained at 37±0.5°C and entire set-up was protected from light.

### Drugs and Chemicals

The drugs and chemicals were obtained from Sigma (St Louis, MO) unless specified otherwise. They were prepared fresh before use. DAF-2DA (Calbiochem, La Jolla, CA) and Calcium Orange- acetoxymethyl ester (AM) (Molecular Probes, Eugene, OR) and nifedipine were dissolved in dimethyl sulfoxide (DMSO). The final bath concentration of chemicals were: apamin (0.3 µM); atropine (1 µM ); calcium orange-AM (10 µM); ω-conotoxin-GVIA (ωCTX) (0.1 µM); 4,5-diaminofluorescein diacetate (DAF-2DA) (10 µM); (100 µM); guanethidine (5 µM); N-ω-nitro-L-arginine methyl ester (L-NAME) (200 µM); nifedipine (1 µM); tetrodotoxin (TTX) (1 µM); and W7 (Calbiochem, La Jolla, CA) (100 µM).

### Dye Loading

Gastric muscle strips were mounted in a chamber and perfused with Krebs' solution prior to loading with dyes/drugs. Calcium orange-AM and/or DAF-2A were added 1 hour prior to EFS. The emission spectrum (576 nm) of Calcium orange can be well resolved from that of DAF-2T (515 nm), thus facilitating simultaneous imaging of the two components.

### Electrical Field Stimulation

Tissues were incubated in fluorescent dyes for one hour prior to EFS and antagonists were applied 20–30 min prior to the dye loading. The EFS was applied under NANC conditions (in the presence of atropine (1 µM) and guanethidine (5 µM)) to block cholinergic and adrenergic responses to elicit nonadrenergic noncholinergic inhibitory responses. The EFS consisted of 3 stimulus trains of 0.5 sec each (square wave pulses of 1 ms at 20 Hz, 70 volts) applied 30 seconds apart. The tissues were immediately mounted on the slides and imaged promptly.

### Imaging with multiphoton microscopy

Dye-loaded and treated tissues after EFS were imaged with BioRad MRC 1024ES multi-photon imaging system (BioRad, Hercules, CA). The imaging system was coupled with a mode-locked titanium∶sapphire laser (Tsunami, Spectra-Physics, Mountain View, CA) operating at 82 MHz repetition frequency, 80 fs pulse duration with a wavelength of 820 nm. Tri/sapphire laser tuned to 820 nm in multi-photon excitation mode at 700 mW was able to excite calcium orange-Ca^2+^ (549 nm) and DAF-2T (495 nm) dyes to consistently generate measurable emissions in orange-red (576 nm) and green (515 nm) regions of the spectrum. The average laser power delivered to the sample was 70–150 mW. Narrow band pass filters were used to separate the emission spectra of the two dyes. A Zeiss Axiovert S100 inverted microscope equipped with a high quality water immersion 40×/1.2 NA, C-apochroma objective was used in the epifluorescence and/or transmission mode to image the nerve varicosities. The 512×512 pixel images were collected in direct detection configuration at a pixel resolution of 0.484 µm with a Kalman-5 collection filter. The nerve varicosities were identified by Z scanning the circular smooth muscle layer and were generally at depths of 150 µm. The images were reconstructed using the BioRad LaserSharp software.

### Relative quantification of Ca^2+^ and NO signals

Impaired nitrergic neurotransmission may be associated with reduced Ca^2+^ response, normal Ca^2+^ response but reduced NO response or normal Ca^2+^ and NO responses to EFS. We determined the relative changes in calcium orange-Ca^2+^ and DAF-NO fluorescence, comparing the intensities of the signals in the antagonist treated, EFS stimulated tissues with the unstimulated control tissues. Three to six boundaries were drawn around arbitrary areas along the nerve varicosities identified by XYZ scanning in a field of view at 320× magnification using an image processor (LaserSharp, BioRad). Fluorescence intensity was integrated over all pixels within the boundary of each individual enclosed area and quantified using LaserSharp (BioRad) and MetaMorph (Universal Imaging, West Chester, PA). The data are presented as the average of at least three blinded experiments performed on different days.

### Immunolabeling with anti-nNOS antibody

For colocalization of NO and nNOS, muscle strips were loaded with the NO indicator, DAF-2DA and EFS applied as described above. The tissues were then fixed in 4% freshly prepared formaldehyde in PBS and were labeled with rabbit anti-nNOS antibody.

### Slow IJP Recordings

Intracellular membrane potential recordings under NANC conditions were made using sharp microelectrodes with high input impedance as described in details in our previous publications [13], [14], [23].

### Statistics

Data were expressed as means±SEM and appropriate tests done to compare significance of difference in means (Student's t test and multiple comparisons, respectively).
